# Editorial: Insights in infectious agents and disease: 2021

**DOI:** 10.3389/fmicb.2023.1239050

**Published:** 2023-07-10

**Authors:** Axel Cloeckaert, Caterina Guzmán-Verri

**Affiliations:** ^1^INRAE, Université de Tours, UMR, ISP, Nouzilly, France; ^2^Programa de Investigación en Enfermedades Tropicales, Escuela de Medicina Veterinaria, Universidad Nacional de Costa Rica, Heredia, Costa Rica

**Keywords:** viruses, bacteria, fungi, virulence, pathogenesis, antimicrobial resistance, epidemiology

The emergence and spread of infectious diseases with pandemic potential occurred trough human history, and were recently reviewed in the section Infectious Agents and Disease by Piret and Boivin (2021). Major pandemics and epidemics involved bacterial infectious diseases such as plague, cholera, or tuberculosis since past centuries, and viral infectious diseases such as flu and the most recently COVID-19 which emerged at the end of 2019, spread globally, and caused millions of deaths. Many infectious diseases leading to pandemics are caused by zoonotic pathogens, originating from an animal source (livestock, wildlife, or companion animals). In addition, some of them may be transmitted to humans through intermediate hosts such as arthropods, which allow them to be grouped as vector-borne and zoonotic infectious diseases.

The present Research Topic, consisting of 36 published articles, provides an overview of recent developments in the field of bacterial, viral, parasitic, and fungal infectious diseases. This overview can be structured according to the type of infectious agent concerned, i.e., mainly bacterial, viral, or fungal, and in the order of importance according to the number of articles published for each infectious agent type. It also reflects the current content of the section Infectious Agents and Disease of Frontiers in Microbiology.

## 1. Bacterial infections

A number of articles dealt with bacterial diversity, epidemiology, and antimicrobial resistance. Gyamfi et al. identified, using VNTR profiling, new *Mycobacterium ulcerans* genotypes in Buruli ulcer endemic communities in Ghana and Côte d'Ivoire. This molecular epidemiology study suggested evidence of possible transmission of *M. ulcerans* from the environment, particularly water bodies from aquatic plants, to humans through open lesions on the skin. Shao et al. reported the phenotypic and molecular characterization of a K54-ST29 hypervirulent *Klebsiella pneumoniae* isolate causing multi-system infection in a patient with diabetes. Community-acquired infection caused by hypervirulent K54 *K. pneumoniae* with diabetes is a particular concern in East Asia. Among zoonotic pathogens, *Streptococcus suis* causes invasive infections in humans and pigs. Kerdsin et al. reported the genomic characterization of *Streptococcus suis* serotype 24 clonal complex 221/234 from human patients. The strains were analyzed and classified according to their antimicrobial resistance genes, pathotyping, virulence-associated gene profiles, and minimum core genome typing. Regarding pathogen transmission, and in particular transmission of tick-borne pathogens, in a hypothesis and theory article, Chang et al. discussed the role of ranged horses in the eco-epidemiology of *Rickettsia raoultii* infection in China. *R. raoultii* gene sequences were indeed detected in both horses and their ticks. In the field of epidemiology, a study of Lang et al., performed in hospitals from Seesen/Germany, reported risk factors of patients with diarrhea for having *Clostridioides* (*Clostridium*) *difficile* infection. Among significant factors identified were the use of diuretics and a diet poor in vegetables.

Five articles of the Research Topic focused on antimicrobial-resistant pathogens and means to combat their emergence and spread. Yao et al. reported the genomic characterization of a uropathogenic *Escherichia coli* ST405 isolate harboring several distinct plasmids carrying *bla*ctx-m antibiotic resistance genes encoding extended-spectrum beta-lactamases. The coexistence of two *bla*ctx-m variants in the same strain increases the risk of the emergence of new *bla*ctx-m variants. Regarding detection of antimicrobial-resistant bacteria, Hayashi-Nishino et al. reported the identification of bacterial drug-resistant cells by the convolutional neural network in transmission electron microscopy images. The proposed method was highly accurate in classifying cells and differentiating between enoxacin-resistant and enoxacin-sensitive cells based on bacterial cell envelope differences. Another bacterial pathogen of concern regarding antibiotic resistance is *Neisseria meningitidis*. Zhang Y. et al. reported the epidemiology of meningococcal disease and carriage, genotypic characteristics and antibiotic resistance of *N. meningitidis* isolates in Zhejiang province, China, during the period 2011–2021. Regarding antimicrobial chemotherapy, a study of Spoto et al. proposed active surveillance cultures and procalcitonin in combination with clinical data to guide empirical antimicrobial therapy in hospitalized medical patients with sepsis. Among possible means to combat the emergence and spread of antimicrobial-resistant microorganisms, Ratia et al. provided a mini-review on gold-derived molecules as new antimicrobial agents. Gold complexes, with their broad-spectrum antimicrobial activities and unique modes of action, appear particularly relevant among several investigated families of derivatives.

Several articles dealt with virulence factors, pathogenicity mechanisms, infection models, immune response, or infectious pathology. Ferrell et al. reviewed the current knowledge on virulence mechanisms of *Mycobacterium abscessus* and their implications for vaccine design. Continued research into *M. abscessus* pathogenesis is of importance for the future development of safe and effective vaccines and therapeutics to reduce global incidence of this emerging pathogen. Cabral et al. reviewed the modulation of the cellular response to *Mycobacterium leprae* and pathogenesis of leprosy. Ménard et al. reviewed the cross-talk between the intestinal epithelium and *Salmonella* Typhimurium, with a focus on the mechanisms developed by *S*. Typhimurium to cross the intestinal epithelium and access to sub-epithelial or systemic sites and survive host defense mechanisms. Schultz M. et al. reported the role of *Staphylococcus aureus* lipoproteins in inducing bone resorption in a mouse model of *S. aureus* septic arthritis. The effect was suggested to be mediated by the lipoproteins' lipid-moiety through monocytes/macrophages. In the field of microbial pathogenesis, Yamazaki et al. reported, using a murine model of infection, that the increased vascular permeability due to spread and invasion of *Vibrio vulnificus* in the wound infection exacerbates potentially fatal necrotizing disease caused by this pathogen. *Helicobacter hepaticus* infection is linked to chronic hepatitis and fibrosis in BALB/c mice. Cao et al. investigated the mechanism underlying the mouse model of *H. hepaticus*-induced hepatocellular carcinoma, and showed that *H. hepaticus* infection promotes the progression of liver preneoplasia via the activation and accumulation of high-mobility group box-1 (HMGB1). Controlled human infection models (CHIMs) have been used to provide invaluable insights in the pathogenesis, host-pathogen interaction and evaluation of vaccines. Sztein and Booth, reviewed CHIMs to fully comprehend the human response to enteric infections, in particular enteric fevers caused by typhoidal *Salmonella* spp., with emphasis on the contributions of CHIMs to uncover the complex immunological responses to these organisms and to provide insights into the determinants of protective immunity.

Regarding infectious pathology, Zimmermann et al. provided additional insight through a case report and review of the literature on reactive arthritis caused by *C. difficile* ribotype 027. This pathology is characterized by the fact that patients suffer from diarrhea or colitis after taking antibiotics, toxigenic *C. difficile* or only the toxins are detectable in the stool and there are no other explanations for the arthritis and diarrhea. Örgel et al. studied during a period of 24 months bacterial colonization and infection rates in patients with transcutaneous osseointegrated prosthetic systems after lower limb amputation. The dominant bacteria identified were Gram-positive bacteria consisting of the following species: *Staphylococcus aureus*, other *Staphylococcus* spp., and *Streptococcus* spp. This study highlighted that the soft tissue inside and around the transcutaneous stoma is colonialized by multiple taxa that changes over time, and a stable Gram-positive dominated bacterial taxa could be a protective factor for ascending periprosthetic infections.

In the field of immune response that concerns both bacterial and viral pathogens, Schultz B. M. et al. reviewed the role of extracellular trap release during bacterial and viral infection. Neutrophil extracellular traps (NETs) are supra-molecular structures produced to kill or immobilize several types of microorganisms, including bacteria and viruses. The contribution of the NET release process to acute inflammation or the prevention of pathogen spreading depends on the specific microorganism involved in triggering this response.

## 2. Viral infections

The COVID-19 disease, caused by the severe acute respiratory syndrome coronavirus 2 (SARS-CoV-2), emerged at the end of 2019 and spread rapidly in human population all over the world becoming the first major pandemic of the 21^th^ century, affecting hundreds of millions people and causing millions of deaths. Several variants have emerged in multiple successive waves during 3 years, leading health and governmental authorities to impose drastic prevention measures, such as lockdown for several months, a situation that was not known previously to the global population, and which resulted in critical economical situations in many countries. On the other hand, this pandemic and associated research has greatly contributed to increase our knowledge regarding all aspects of a viral infectious disease, and to develop rapidly new diagnostic, therapeutic, prevention, and vaccine strategies.

Markarian et al. reviewed the identifying markers of emerging SARS-CoV-2 variants in patients with secondary immunodeficiency. Immunodeficient patients are one of the vulnerable cohorts that are the most susceptible to this virus, and chronic infections in the presence of anti-COVID-19 treatments may potentially lead to the evolution of the virus in the host. Amino acid modifications identified occurred in a variety of viral proteins, including those involved in pathogenesis such as spike proteins. Some of them were identified as recurrent *de novo* changes that can potentially play in SARS-CoV-2 pathogenesis and evolution. Garanina et al. studied antibody and T cell immune responses to SARS-CoV-2 peptides in COVID-19 convalescent patients. Their data highlighted how humoral immune responses and cytotoxic T cell responses to some of these peptides could contribute to SARS-CoV-2 pathogenesis. Regarding SARS-CoV-2 transmission, Huang et al. reported an integrated analysis revealing the characteristics and effects of SARS-CoV-2 maternal-fetal transmission. SARS-CoV-2 spreads by air with a transmission rate of up to 15%, but the probability of its maternal-fetal transmission through the placenta is reported to be significantly lower at around 3%. Cellular and viral-associated factors contributing to this transmission were investigated in the study of Huang et al..

In terms of vaccine candidates, Liu et al. reported that bacterially (*E. coli*)-expressed SARS-CoV-2 receptor binding domain fused with cross-reacting material 197A-domain elicits high level of neutralizing antibodies in mice. This type of vaccine candidate was developed as a less expensive and accessible vaccine option (relative to the mostly used DNA-based vaccines for example), particularly for developing countries. Zhang L. et al. evaluated an oral SARS-CoV-2 spike protein recombinant yeast candidate and showed that this vaccine candidate prompted specific antibody and gut microbiota reconstruction in mice. Besides respiratory issues, some COVID-19 patients have gastrointestinal symptoms and intestinal flora dysbiosis. The use of the yeast recombinant candidate could fulfill both the agent-driven immunoregulation and gut microbiome reconstitution (as probiotic). In another study, Zhang H. et al. assessed two inactivated vaccines against SARS-CoV-2 for inducing neutralizing antibody and their level of persistence in a Chinese population of vaccine recipients. Neutralizing antibody levels persisted for only 6 months after the second dose of vaccine, and therefore a third vaccine dose was recommended. In addition, Zhuang et al. reviewed the protection duration of COVID-19 vaccines. With a continued adaptation of SARS-CoV-2 to transmission and concomitant immune escape, the authors concluded on the urgency to make continued efforts to optimize vaccine, implement prime and booster campaigns, and further explore multiple and additional layers of protection against infection. It is also highly important to monitor the evolution and mutation patterns of SARS-CoV-2, to establish a forecasting model for viral mutations. The knowledge on the characteristics of future variants may enable shortened timelines to vaccine and therapeutic drug development and help in the control of future COVID-19 outbreaks.

In relation with COVID-19 and other viral or bacterial infections, Velappan et al. proposed in a perspective article warning signs of potential black swan outbreaks in infectious disease. Black swan events in infectious disease describe rare but devastatingly large outbreaks.

Regarding viral, bacterial, or parasitic pathogen transmission, Guo et al. reviewed the association of common zoonotic pathogens with concentrated animal feeding operations (CAFOs). Animal farming has intensified significantly in recent decades, with the emergence of concentrated animal feeding operations in industrialized nations. This led to heavy environmental contamination with pathogens which promoted the emergence of hyper-transmissible and virulent pathogens. Among them were highly pathogenic avian influenza viruses, hepatitis E virus, *E. coli* O157:H7, *S. suis*, methicillin-resistant *S. aureus* (MRSA), and *Cryptosporidium parvum*. The authors conclude on control measures that should be developed to slow down the dispersal of zoonotic pathogens associated with CAFOs and prevent the emergence of new pathogens of epidemic and pandemic potential.

## 3. Fungal infections

Acosta-España and Voigt provided a mini-review on risk assessment, clinical manifestation, prediction, and prognosis of mucormycosis and their implications for pathogen- and human-derived biomarkers. Mucormycosis is a fungal disease caused by members of the fungal order Mucorales, with various clinical pictures, ranging from rhinocerebral via pulmonary to gastrointestinal forms and occurs especially in immunocompromised hosts. A major issue is misdiagnosis with other infections, whilst rapid and precise diagnosis is mandatory because symptoms are non-specific and the disease can rapidly progress with often a fatal outcome. Therefore, this mini-review explores several possibilities to address or prevent the misidentification issues, such as early prediction of host susceptibility to mucormycosis using single nucleotide polymorphisms in human host genes together with risk assessment and early diagnosis to reduce mortality in patients suffering from mucormycosis. Dogra et al. provided another mini-review on mucormycosis in the context of the COVID-19 crisis, focusing on pathogenesis, diagnosis, and novel treatment strategies to combat the spread. The COVID-19 pandemic has indeed paved way for secondary ominous fungal infections like mucormycosis. This mini-review summarizes current and imminent approaches that could aid effective management of such secondary infections in a global pandemic situation. The authors propose the development of new antifungal drugs, antimicrobial peptides, and nanotechnology-based approaches for drug delivery to help combat this infection and curb its spread. Another fungal disease affecting immunocompromised hosts is reported by Taverne-Ghadwal et al., with the epidemiology and prevalence of oral candidiasis in HIV patients from Chad in the post-HAART (highly active antiretroviral therapy) era. The authors studied the prevalence of several *Candida* species and showed that the prevalence of oral candidiasis was significantly higher in untreated than in HAART-treated HIV-positive patients. The species distribution was similar to other countries around the world, with *C. albicans* being dominant. HAART therapy reduced oral candidiasis caused by *C. albicans* and led to a species shift toward non-*albicans Candida* species.

In addition to studies or reviews dedicated to the specific pathogens cited above, Martynova et al. reviewed the inflammasome contribution to the activation of Th1, Th2, and Th17 immune responses. Inflammasomes are cytosolic polyprotein complexes formed in response to antigens of all infectious agent types. Regarding immune responses, Arch et al. provided a review on *Drosophila melanogaster* as a model to study innate immune memory. This non-mammalian model as been widely used for innate immune research since it naturally lacks an adaptive response.

Two articles of this Research Topic dealt also with the importance of microbiota in infection and infectious pathology. Negi et al. provided a mini-review on neonatal microbiota-epithelial interactions that impact infection. Wang et al. reviewed the role of the microbiome in male infertility, including its association with microbial infection in the genital tract.

## Author contributions

All authors listed have made a substantial, direct, and intellectual contribution to the work and approved it for publication.
